# Influence of intraoperative electromyographic changes and surgical extent on post-thyroidectomy esophageal motility: a prospective cohort study

**DOI:** 10.3389/fendo.2025.1651936

**Published:** 2025-10-06

**Authors:** Mehmet Gunay, Yalin Iscan, Ismail Cem Sormaz, Nihat Aksakal, Fatih Tunca, Filiz Akyuz, Yasemin Giles Senyurek

**Affiliations:** ^1^ Department of General Surgery, Istanbul Faculty of Medicine, Istanbul University, Istanbul, Türkiye; ^2^ Department of Internal Medicine, Istanbul Faculty of Medicine, Istanbul University, Istanbul, Türkiye

**Keywords:** thyroidectomy, ıntraoperative neuromonitoring, upper esophageal sphincter resting pressure, high-resolution manometry, esophageal motility

## Abstract

**Background:**

Thyroidectomy may lead to postoperative swallowing difficulties. This study aimed to evaluate the relationship between electromyographic (EMG) changes observed during intraoperative neuromonitoring (IONM) and swallowing problems following lobectomy, total thyroidectomy (TTx), and total thyroidectomy with central lymph node dissection (TTx+CLND), as assessed using high-resolution manometry (HRM).

**Methods:**

This prospective study included 36 patients who underwent thyroid lobectomy (n=9), TTx (n=15), or TTx+CLND (n=12) by using IONM. All patients were questioned about dysphagia and underwent HRM preoperatively and at postoperative 6th month. Demographic data, the dominant nodule size, total thyroid volume, IONM findings and HRM results were recorded. The pre-and postoperative changes in HRM results were evaluated according to the clinical characteristics, extent of surgery and IONM data.

**Results:**

Five (14%) reported *de novo* postoperative dysphagia. No loss of signal during IONM and postoperative vocal cord palsy were observed. Adverse EMG changes and combined event (CE) occurred in 8 (22%) and 2 (5.5%) patients, respectively. Postoperative upper esophageal sphincter resting pressure (UESRP) significantly decreased from a median of 181 mmHg (range: 12–407 mmHg) to 109 mmHg (range: 8.6–407 mmHg), postoperatively (p=0.03). The percentage of UESRP decrease was 64% (range: -82_20%) and 25% (range: -86 _135%) in patients with and without postoperative *de novo* dysphagia (p=0.06). In the TTx group, which had significantly larger tumor size and higher thyroid volumes compared to the TTx+CLND group, UESRP decreased by 44%, versus 16% in the TTx+CLND group (p=0.001). Patients who experienced adverse EMG changes exhibited a significantly lower median percentage decrease in UESRP (-3%, range: -54% to 52%) than those without EMG abnormalities (-34%, range: -86% to 135%) (p=0.04). No significant correlation was found between the EMG changes in external branch of superior laryngeal nerve and postoperative UESRP alterations.

**Conclusion:**

This prospective clinical study demonstrated that UESRP decreases after thyroidectomy even in patients without IONM evidence of nerve injuries. Patients with large nodules, high thyroid volume, and new-onset dysphagia tended to show greater reductions in UESRP, whereas those with intraoperative adverse EMG changes exhibited less pronounced decreases. These heterogeneous responses may reflect complex neuromuscular adaptations involving the cricopharyngeus muscle.

## Introduction

Thyroidectomy is one of the most commonly performed endocrine surgeries worldwide (1). Post-thyroidectomy morbidities significantly affect patients’ quality of life and have medicolegal implications for surgeons. Patients frequently report alterations in voice and swallowing functions after thyroidectomy, even without laryngeal nerve damage (2, 3). The recurrent laryngeal nerve (RLN) and the superior laryngeal nerve (SLN) *play essential roles* in phonation and swallowing. The cricopharyngeus muscle controls deglutition at the level of the upper esophageal sphincter (UES*), while intrinsic laryngeal muscles regulate vocalization* (4). The internal branch of the SLN innervates the supraglottic area and vocal cords; injury causes dysphagia and aspiration (4, 5). The external branch of the SLN (EBSLN) is the motor nerve of the cricothyroid (CT) muscle, producing vocal cord tension (6). Unilateral recurrent nerve injury during thyroidectomy causes voice problems in all patients but swallowing problems in about 30% (2, 7, 8).

The role of central lymph node dissection (CLND) in increasing RLN damage risk is controversial (9–14). While vocal cord innervation occurs after laryngeal entry, esophageal nerve branches are essential for swallowing. Incomplete glottic closure may be associated with swallowing disorders, potentially due to damage to esophageal branches of the RLN. CLND may disrupt these nerve connections during adipose tissue removal, impairing swallowing functions.

Intraoperative nerve monitoring (IONM) is increasingly used to reduce RLN injury risk during thyroidectomy. Vasileiadis et al. (15) *reported that IONM serves as an independent protective factor against nerve damage*. Decreased nerve stimulation threshold amplitude and prolonged latency during dissection indicate potential nerve damage, allowing intraoperative technique modification to prevent injury (16).

Despite the growing use of IONM, *there is a paucity of objective research* evaluating its relationship with post-thyroidectomy swallowing impairment. This study aimed to evaluate the relationship between electromyographic (EMG) changes observed during IONM and swallowing problems following lobectomy, total thyroidectomy (TTx), and total thyroidectomy plus CLND (TTx+CLND), as assessed by using high-resolution manometry (HRM).

## Methods

From March 2021 to December 2022, patients aged 20 to 80 years who were scheduled to undergo thyroid lobectomy, total thyroidectomy (TTx), or TTx with central lymph node dissection (CLND) for nodular thyroid disease or papillary thyroid cancer (PTC) were considered eligible. Exclusion criteria were: prior neck surgery, history of neck radiotherapy, severe thyroiditis, hyperthyroidism, pre- or postoperative vocal cord paralysis, significant cardiopulmonary conditions, refusal to participate, diabetes or neuromuscular ailments, and use of medications impacting esophageal motility. Among the 40 initially enrolled patients, 3 declined postoperative manometry due to discomfort. One patient with locally invasive thyroid cancer was excluded due to intraoperative unilateral laryngeal nerve injury; thus, 36 patients were included.

All patients underwent fiber-optic laryngoscopy before and within the first 72 hours after surgery. Postoperative laryngoscopic examination was scheduled in this period to minimize the potential effects of transient intubation-related laryngeal edema and mucosal irritation, which could interfere with the accurate assessment of vocal cord mobility. Preoperative neck ultrasonography (USG) was performed, recording the maximum diameter of the dominant nodule. Thyroid volume was calculated using the formula height × width × length × 0.479, defined by Brunn et al. (17) and accepted by the World Health Organization.

All surgeries were performed under 2.5x magnification loupe using continuous or intermittent IONM. Equipment setup, anesthesia, IONM standards, EMG definitions, and data interpretation followed International Intraoperative Neural Monitoring Study Group (INMSG) guidelines (18). Response evaluation of the vagus nerve and RLN was performed at the beginning (V1,R1) and end of the operation (V2,R2). Pre- and post-dissection EMG activity was recorded in the EBSLN’s (S1 and S2) in all procedures (19). Adverse EMG parameters were defined as amplitude decrease ≥50% or latency increase ≥10% from baseline. A combined event (CE) was identified as concurrent amplitude decrease >50% and latency increase >10%. Surgical maneuvers were adjusted upon observing adverse EMG changes, with traction released to allow recovery. Recovery was defined as amplitude reaching >50% of initial baseline. LOS was defined as amplitude <100 μV.

Serum calcium and parathyroid hormone (PTH) levels were monitored on the first postoperative day and those with low levels of serum calcium or PTH received calcium and vitamin D supplements. All patients underwent HRM to evaluate swallowing function within one week before and six months after surgery. This timing was chosen to minimize the confounding effects of transient wound healing, postoperative edema, and inflammatory changes, and to evaluate long-term, stable esophageal motility alterations. HRM was performed by a gastroenterologist blinded to the surgical procedure using the Unisensor UniTip High Resolution catheter (MMS, Netherlands) with 36 pressure sensors. Patients were asked to fast for at least 8 hours before the procedure. Chicago classification version 4.0 was used for the manometry protocol (20). All patients were verified to be in the euthyroid and euparathyroid states at the time of HRM procedure. In addition, patients asked about dysphagia complaints during pre- and post-operative HRM exams. Dysphagia was evaluated preoperatively and postoperatively by structured questioning. Patients were asked about symptoms such as pain, discomfort, cough or choking while swallowing, a sensation of a lump or foreign body in the throat, and the feeling of food being stuck. Dysphagia can be present even before surgery; therefore, both pre- and postoperative symptoms were documented. No validated questionnaire was used. The postoperative follow-up was at least 6 months, with the last visit in June 2023. The study was approved by the Istanbul Medical Faculty Clinical Research Ethics Committee (Approval no: 2020/1838).

### Statistical analysis

Continuous variables were summarized as means, standard deviations, medians, and ranges. Student’s t-test, Mann-Whitney U-test, and Kruskal-Wallis test were used to compare continuous variables between groups for normally and non-normally distributed data. Wilcoxon’s signed rank test was used to compare values from the same group at different time points. Univariate analysis by chi-square test and Fisher’s exact test examined differences in proportions. A two-tailed p-value < 0.05 was considered statistically significant. Analyses were performed using the Statistical Package for the Social Sciences version 22 for Windows (SPSS, IBM Inc., Chicago,IL, USA).

## Results

### Patient characteristics and the extent of surgery

Of the 36 patients, 25 (69%) were women and 11 (31%) were men, with a median age of 46.5 years (range 20-73). For most patients (n=21, 58%), surgery was indicated by Bethesda 5 or 6 cytology on fine-needle aspiration biopsy of a thyroid nodule, while 15 patients (42%) had surgery for nodular goiter causing compressive symptoms. Retrosternal extension of the thyroid gland was observed in 10 patients (28%). The mean diameter of the solitary or dominant thyroid nodule was 2.37 ± 1.3 cm. The median volumes of the total thyroid glands, right lobes, and left lobes were 22 cm³ (range: 9.8–458 cm³), 11.9 cm³ (range: 4.5–258 cm³), and 11 cm³ (range: 4.3–200 cm³), respectively.

Of the 36 patients, 15 (42%) underwent TTx, 12 (33%) TTx+CLND, and 9 (25%) lobectomy. Intermittan or continuous IONM were used in 20 (56%) and 16 (44%) patients, respectively. Demographic data, nodule size, thyroid volume, and retrosternal extension rates in the lobectomy, TTx, and TTx+CLND groups are summarized in **Table 1**. Age and sex showed no significant difference between the groups. The dominant nodule size was significantly larger in the TTx group than in the TTx+CLND group (3.1 ± 1.4 *vs* 1.5 ± 0.46; p=0.001). Patients in the TTx group had significantly higher total thyroid volume compared to the lobectomy and TTx+CLND groups (p=0.01 and p=0.0001, respectively) (**Table 1**).

**Table 1:** Characteristics of patients according to the extent of surgery.

Variables	L (n=9)	TTx (n=15)	TTx+CLND (n=12)	L*vs* TTx	L*vs* TTx+CLND	TTx*vs* TTx+CLND
Age (mean ± SD)	48.2 ± 9.1	49.2 ± 11.3	43 ± 15.8	0.8	0.4	0.2
Female/male	6/3	11/4	8/4	0.9	0.9	0.9
Nodule diameter (cm, mean ± SD)	2.3 ± 1.5	3.1 ± 1.4	1.5 ± 0.46	0.2	0.1	0.001 *
Thyroid volume (median, range cm³)	17.9 (9.8–40)	47 (16–458)	18.4 (10–28)	0.01*	0.8	0.0001*
Retrosternal extension n (%)	3 (37.5)	7 (46.6)	0 (0)	1	0.04*	0.007*

Values are presented as mean ± SD, median (range), or n (%). Statistical significance indicated by *p < 0.05.

CLND, Central lymph node dissection; L, Lobectomy; TTx, Total thyroidectomy.

### Intraoperative EMG findings

No significant difference was found between baseline and final EMG parameters (**Table 2**). None of the patients experienced loss of signal during the operation. Reversible adverse EMG changes were observed in eight (22%) patients (four in TTx+CLND, two in TTx, and two in lobectomy). In another two (5.5%) patients, CE occurred on the left side during TTx+CLND. The left V2 amplitude decreased by 52% and 64%, with a latency increase of more than 10% of the baseline in these two patients. However, recovery in EMG parameters was obtained within 20 minutes after the release of traction in these two patients. Comparing pre- and post-dissection IONM data of EBSLN’s, post-dissection adverse EMG parameters were observed in nine (25%) patients.

**Table 2:** Presurgical dissection and postsurgical dissection amplitude and latency values of the nerves.

IONM parameter	Amplitude (μV) (pre-dissection)	Amplitude (μV) (post-dissection)	P	Latency (mS) (pre-dissection)	Latency (mS) (post-dissection)	P
Right V	0.50 ± 0.35	0.49 ± 0.34	0.6	3.97 ± 0.77	4.00 ± 0.94	0.6
Right R	0.52 ± 0.27	0.57 ± 0.33	0.2	1.92 ± 0.50	1.91 ± 0.60	0.975
Right S	0.21 ± 0.28	0.23 ± 0.29	0.3	1.65 ± 0.40	1.65 ± 0.43	0.934
Left V	0.50 ± 0.32	0.49 ± 0.31	0.864	6.04 ± 1.30	6.37 ± 0.83	0.08
Left R	0.77 ± 0.56	0.76 ± 0.49	0.829	2.21 ± 0.47	2.21 ± 0.57	1.000
Left S	0.14 ± 0.15	0.17 ± 0.16	0.43	1.64 ± 0.25	1.70 ± 0.31	0.371

Values are presented as mean ± SD. p < 0.05 was considered statistically significant.

Pre-dissection corresponds to V1/R1/S1, post-dissection corresponds to V2/R2/S2 stimulation.

IONM, Intraoperative neuromonitoring; R, Recurrent laryngeal; S, External branch of superior laryngeal nerve; V, Vagus; µV, microvolts; mS, milliseconds.

### Postoperative results

Postoperative histopathological examination showed papillary thyroid carcinoma in 29 patients (80.5%), with a mean tumor diameter of 1.3 ± 0.96 cm. Papillary microcarcinomas made up 45% (n=13) of papillary thyroid cancers. Postoperative indirect laryngoscopy showed no vocal cord paralysis in any of the patients. Transient hypoparathyroidism occurred in 12 patients (33%) who received postoperative calcium and vitamin D supplementation. Serum calcium and PTH levels normalized within 2 to 4 weeks postoperatively in these patients.

### Evaluation of swallowing, HRM results and IONM data

Of the 36 patients, 14 (39%) experienced dysphagia both before and after the operation, while 17 (47%) did not exhibit dysphagia preoperatively or postoperatively. The remaining five (14%) patients reported postoperative dysphagia onset that was absent prior to surgery. After thyroidectomy, UESRP decreased in most patients (n=25, 69%) and increased in 11 (31%) compared to preoperative measurements. Overall, UESRP significantly decreased from 181 (12–407) to 109 (8.6-407) mmHg post-surgery (p=0.03) (**Table 3**). The percentage change in UESRP was -27.4%, ranging from an 86% decrease to a 135% increase. Other motility parameters, including lower esophageal sphincter pressure, distal latency, UES integrated relaxation pressure, peristaltic breaks, and distal contractile integral did not change significantly post-surgery (**Table 3**). UESRP was the only HRM parameter showing significant changes pre- and post-surgery; thus, its correlation with age, sex, dysphagia complaints, thyroid volume, nodule diameter, and extent of surgery was further investigated.

**Table 3:** High-resolution manometry parameters in patients before and after thyroidectomy.

HRM parameters	Before surgery	After surgery	P-value
UES resting pressure (mmHg)	181 (12–407)	109 (8.6–407)	0.03*
UES IRP 0.2 sec (mmHg)	5.2 (0.2–86)	5 (0.1–70.8)	0.82
UES IRP 0.8 sec (mmHg)	26.8 (5–142)	19 (3–160)	0.08
LES resting pressure (mmHg)	18.5 (6.7–62)	18.5 (3.2–52)	0.85
LES IRP (mmHg)	6.35 (0.5–24.3)	5.1 (0.2–21.5)	0.451
DCI (mmHg·s·cm)	345 (56–2925)	502 (75–2251)	0.88
DL (s)	8 (5–82)	8.3 (5–65)	0.94
Peristaltic breaks (cm)	5.8 (0–15.9)	7.5 (0–17.5)	0.55

Values are presented as median (range). *p < 0.05 was considered statistically significant.

DCI, Distal contractile integral; DL, Distal latency; HRM, High-resolution manometry; IRP, Integrated relaxation pressure; LES, Lower esophageal sphincter; UES, Upper esophageal sphincter.

**Figure 1:**
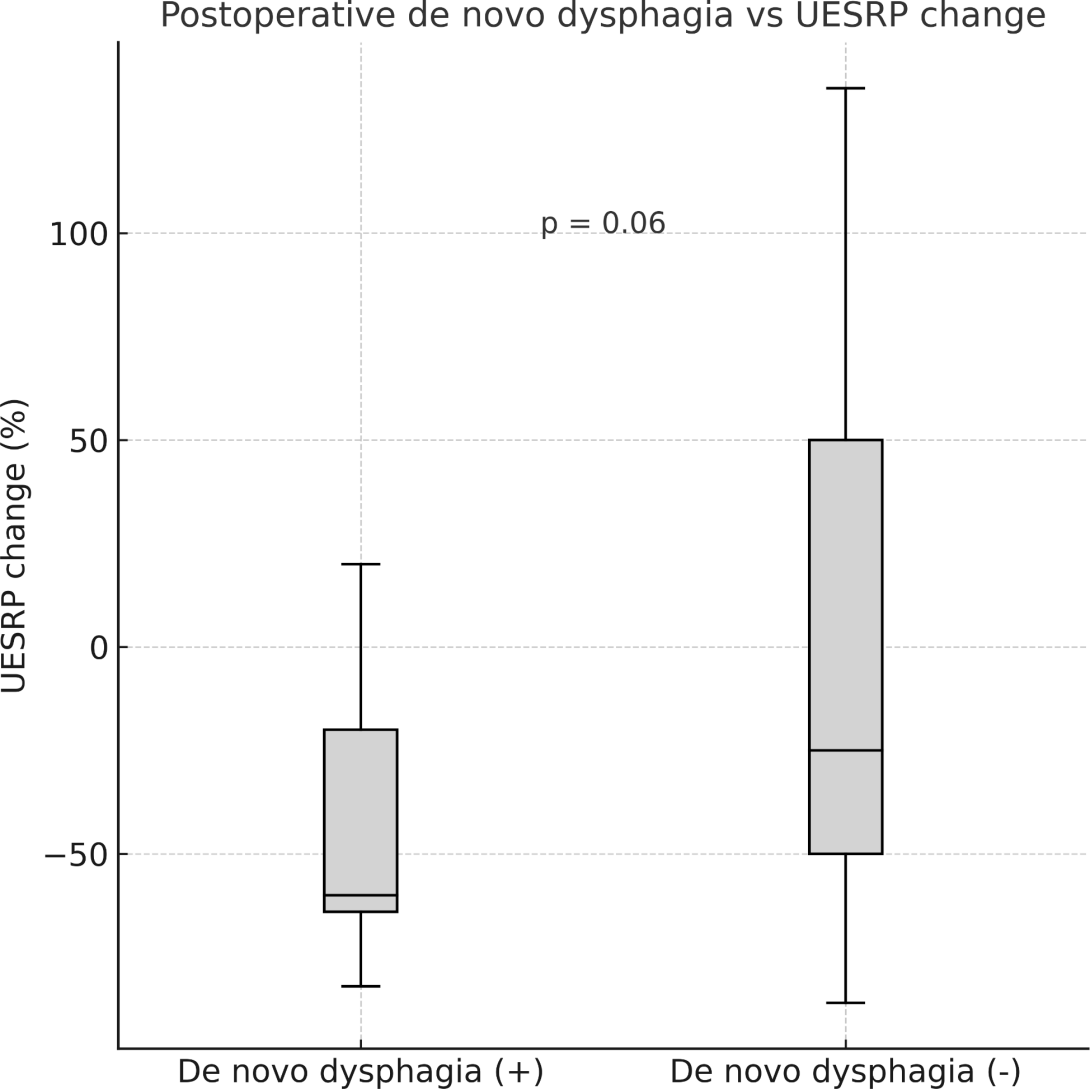
Median postoperative change in upper esophageal sphincter resting pressure (UESRP) in patients with (+) and without (–) postoperative *de novo* dysphagia (p = 0.06).

**Figure 2:**
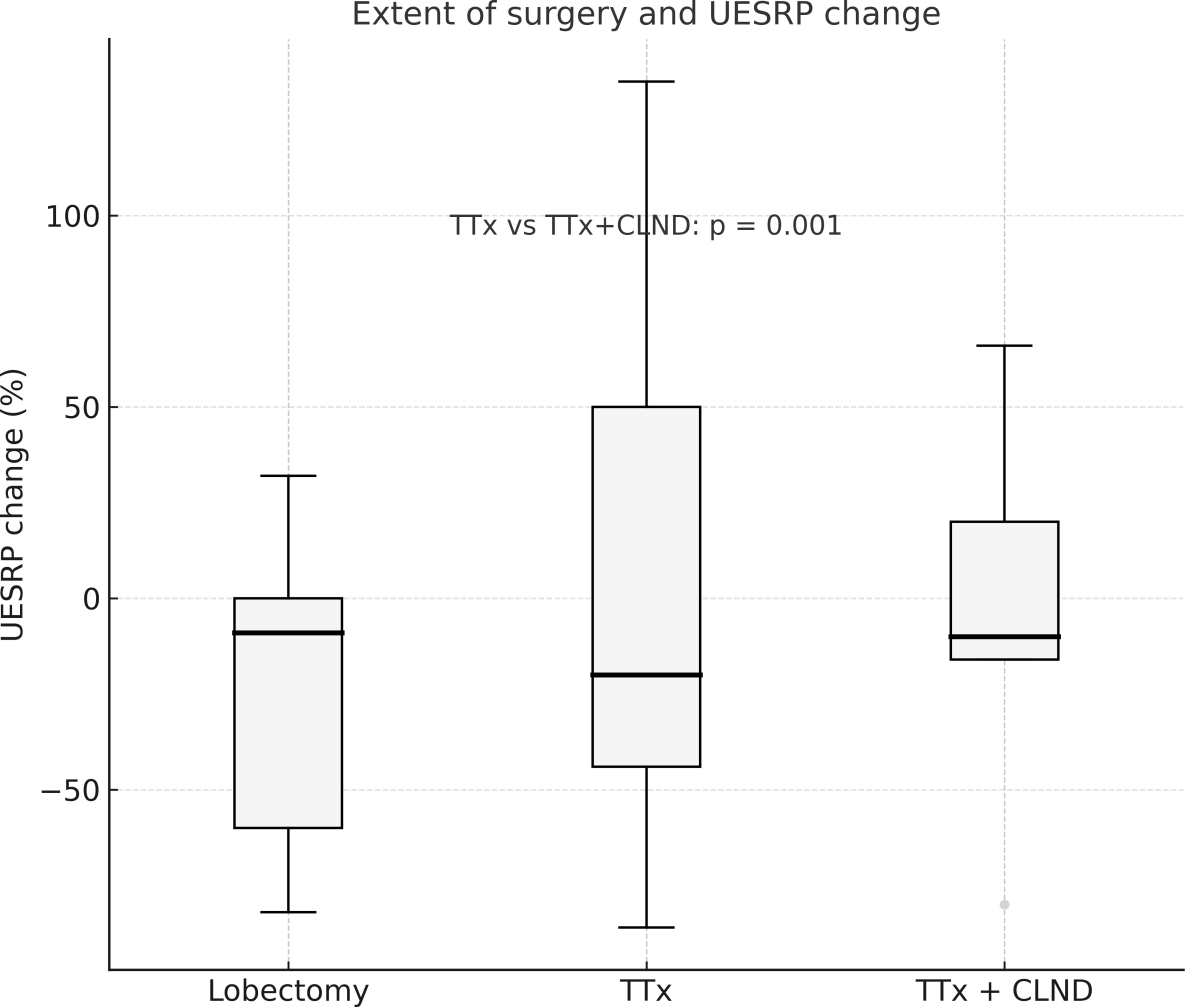
The percentage change in UESRP according to the extent of surgery. Median UESRP decreases in the TTx, TTx+CLND, and lobectomy groups were 44%, 16%, and 8.7%, respectively (p=0.004). A significant difference was found only between the TTx and TTx+CLND groups by pairwise comparison (44% *vs*. 16%; p=0.001). L, Lobectomy; TTx, Total thyroidectomy; TTx CLND, Total thyroidectomy+CLND; UESRP, Upper esophageal sphincter resting pressure.

No significant differences in demographics, dysphagia complaints, nodule size, thyroid volume, or surgery extent were found between patients with decreased or increased postoperative UESRP (**Table 4**). In patients with dysphagia, both pre- and postoperatively, the median UESRP change was -11% (range: -86 to 135%), whereas it was -27.6% (range: -82 to 53%) in those with no dysphagia pre- and postoperatively (p=0.3). In the five patients presenting *de novo* dysphagia, the UESRP significantly decreased by 64% (range: -82_20%), while in the 31 patients without postoperative changes related to dysphagia, the decrease was 25% (range: -86 _135%) (p=0.06) (**Figure 1**). No adverse EMG parameters or CE were observed by IONM data in the five patients with *de novo* dysphagia. Preoperative and postoperative UESRP changes were not significant in the lobectomy, TT, and TTx+CLND groups, individually (**Table 5**). Median UESRP decreases in the TTx, TTx+CLND, and lobectomy groups were 44%, 16%, and 8.7%, respectively (p=0.004). Pairwise comparison revealed a significant difference only between the TTx and TTx+CLND groups (44% *vs*. 16%; p=0.001). No significant difference was observed between the lobectomy and TTx groups or between the lobectomy and TTx+CLND groups (p=0.6 and p=0.5, respectively) (**Figure 2**). In patients with and without reversible adverse EMG parameters, the median UESRP change was -3% (-54% to 52%) and -34% (-86% to 135%), respectively (p=0.04). In two patients with CE, postoperative UESRP increased by 15.3% in one and decreased by 16.6% in the other. The median postoperative UESRP decrease in these two patients with CE was 0.67%, whereas, it was 27% (range -86%_135%) in those without CE (p=0.4). In patients with and without adverse EMG parameters in EBSLN, the median postoperative UESRP change was similar (-27.6% (-82_53) *vs* -25.7% (-86_135), respectively; p=0.4) (**Table 6**).

**Table 4:** Comparison of demographic data, clinical characteristics and the extent of surgery in patients with postoperatively decreased and increased UESRP.

Postoperative UESRP			
Variables	Decreased (n=25)	Increased (n=11)	P-value
Age (yrs) mean ± SD	48 ± 12.4	44 ± 12.8	0.4
Female/male	16/9	9/2	0.4
Nodule size (cm) mean ± SD	2.4 ± 1.4	2.2 ± 1.3	0.73
Thyroid volume (cm³), Median (range)	21 (10–458)	22 (9.8–72)	0.9
With pre-post op dysphagia TTx (%)	8 (32)	6 (54.5)	0.4
With *de novo* dysphagia (%)	4 (16)	1 (9.1)	0.4
Without pre-post op dysphagia (%)	13 (52)	4 (36.4)	0.4
Lobectomy (%)	6 (24)	3 (27)	0.5
TTx (%)	12 (48)	3 (27)	0.5
TTx+CLND (%)	7 (28)	5 (45)	0.6

Values are presented as mean ± SD, median (range), or n (%). p < 0.05 was considered statistically significant.

CLND, Central lymph node dissection; TTx, Total thyroidectomy; UESRP, Upper esophageal sphincter resting pressure.

**Table 5:** Preoperative and postoperative measurements and the postoperative percentage change in UESRP following thyroid surgery.

Variables	UESRP (mmHg) median (range)	% change in UESRP median (range)	P-value
Lobectomy	Preop: 249 (47–407) Postop: 124 (46–386)	-8.7 (–82–32)	0.1
TTx	Preop: 169 (12–357) Postop: 95 (9–406)	-44 (–86–135)	0.1
TTx+CLND	Preop: 118 (32–364) Postop: 126 (16–328)	-16 (–80–66)	0.3

Values are presented as median (range). p < 0.05 was considered statistically significant.

CLND, Central lymph node dissection; TTx, Total thyroidectomy; UESRP, Upper esophageal sphıncter resting pressure.

**Table 6:** The percentage change in UESRP in patients with and without adverse EMG changes and CE according to the IONM data.

Variables	UESRP change% (median)	P
Adverse EMG changes (vagus) Yes (n=8) No (n=26)*	-3(-54_52) -34(-86_135)	0.04 *
Adverse EMG changes (EBSLN) Yes(n=9) No(n=27)	-27,6(-82_53) -25.7 (-86_135)	0.4
CE Yes (n=2) No (n=34)	-0.67(-17_15.3) -27(-86_135)	0.4

Values are presented as median (range). *p < 0.05 was considered statistically significant.

*Two patients with CE not included.

CE, Combined event; EBSLN, External branch of superior laryngeal nerve; EMG, Electromyography; IONM, intaroperative neuromonitoring; UESRP, upper esophageal sphıncter resting pressure.

## Discussion

Swallowing difficulties, particularly dysphagia, are common in patients after thyroid surgery (21). Injuries to the inferior laryngeal nerve and EBSLN during thyroid surgery can cause voice and swallowing impairments (22). Many patients report dysphagia even after uncomplicated thyroidectomy without any nerve injury (21, 23). Swallowing impairments resolve within 6 months after thyroid surgery for most patients, but some have symptoms beyond 12 months (1, 24). Possible mechanisms of post-thyroidectomy dysphagia include arytenoid trauma from intubation, reduced laryngeal movements due to laryngotracheal adhesions, and damage to nerves innervating the pharynx and anastomoses between the SLN and RLN (2, 3, 7, 25).

Numerous methods, including quantitative and diagnostic tools, have been used to evaluate voice and swallowing impairment after thyroid surgery (26, 27). Few studies have investigated swallowing problems using objective methods, such as USG, video endoscopy, electrophysiological examinations, video fluoroscopy, video strobolaryngoscopy, and esophageal manometry (28). Several studies analyzed changes in laryngeal or hyoid bone mobility post-thyroidectomy and their impact on swallowing. Although laryngeal movement significantly reduced after surgery, no significant correlation was detected between laryngeal mobility and swallowing difficulties (29). Similarly, another study did not find a significant association between thyroidectomy and hyoid bone mobility (30).

The incidence of postoperative dysphagia appears to be influenced by extent of thyroidectomy, with more extensive procedures potentially increasing risk. Some studies reported higher dysphagia prevalence after TTx than lobectomy (24, 31). Patients undergoing lymph node dissection also show increased swallowing disorders (24, 31–33). However, other studies found no correlation between postoperative swallowing impairment and the extent of thyroidectomy (34, 35). In our study, five patients without prior dysphagia exhibited onset of dysphagia after surgery and three of them underwent TTx or TTx +CLND. We also observed that the postoperative reduction ratio of UESRP was lowest in the lobectomy group and highest in the TTx group.

Although questionnaires about swallowing symptoms before and after thyroidectomy provide knowledge on surgery’s impact on swallowing, these tests are subjective. Several objective studies have assessed thyroid surgery’s effect on esophageal motility. Scerrino et al. (36) used conventional water manometry to investigate esophageal motility changes and their association with voice and swallowing impairment after thyroidectomy. Their study included 36 patients who underwent total thyroidectomy, excluding those with thyroid malignancy needing lymph node dissection, thyroid volume > 60 ml, severe thyroiditis, hyperthyroidism, previous neck surgery, and pre- or postoperative RLN palsy. Preoperative swallowing problems were recorded in 78% of patients, improving 35–40 days after surgery. However, swallowing problems worsened or developed *de novo* in 20% of patients after surgery. The authors found that UESRP significantly decreased in the entire group after thyroidectomy, most prominently in patients with *de novo* UES incoordination. In our study, we also observed a significant UESRP decrease in the whole group, with a significantly higher reduction ratio in patients developing dysphagia after thyroidectomy than those with unchanged swallowing status postoperatively.

Sorensen et al. (37) evaluated thyroidectomy’s effect on swallowing symptoms and esophageal motility by assessing goiter symptoms and using HRM before and after surgery in 33 patients with benign nodular goiter. The median thyroid weight removed was 50 g (range: 8-607), and 64% of the patients underwent thyroid lobectomy. No correlation was found between symptoms and manometry results. They reported an overall postoperative increase in UES basal pressure at 6 months; however, regression analyses demonstrated a greater reduction in UES basal pressure and peak pressure 4 cm below the UES in total thyroidectomy patients compared with those with lobectomy or isthmectomy. They suggested UES closing pressure might be reduced due to compression in large goiters and postoperative tissue fibrosis might contribute to reduced UES pressure. In our study, the greatest postoperative UESRP reduction was in the total thyroidectomy group, with larger thyroid volume and nodule size than in lobectomy and TTx+CLND groups. Two prospective studies using conventional and high-resolution manometry found no correlation between esophageal motility and swallowing impairment after thyroidectomy (36, 37). This might be due to inadequate statistical power from the limited sample size. Taken together, these findings highlight that postoperative UESRP responses can vary substantially, reflecting heterogeneous or compensatory cricopharyngeal mechanisms. IONM is increasingly used in thyroid surgery. Most studies evaluating post-surgery swallowing functions via questionnaires or objective methods did not employ IONM (24, 29–38). Pereria et al. (7) compared the long-term prevalence of upper aerodigestive symptoms in patients who underwent uncomplicated thyroidectomy with age and sex matched patients who underwent laparoscopic cholecsytectomy. They observed higher prevalence of long-term swallowing difficulties in thyroidectomized patients. The authors believe this may have resulted from injuries to minor sensory branches of the RLN, as no patients had clinical evidence of RLN injury. However, routine IONM and postoperative vocal cord examination were not performed in their cohort. Both RLN and EBSLN injuries impair voice and swallowing functions, and it is not possible to verify the functional integrity of these nerves during thyroidectomy without using IONM. Lower prevalence of long term upper aerodigestive symptoms were observed in patients who underwent thyroidectomy by using IONM compared to those operated without IONM, despite identical surgical techniques in both groups (39).

However, early postoperative swallowing problems are frequent despite postoperative normal vocal cord thyroarytenoid and CT muscle functions. In a prospective randomized study by Lombardi et. (23) investigated swallowing impairment after thyroid surgery in patients who underwent laryngeal EMG at the postoperative first month to examine the thyroarytenoid and CT muscles in order to evaluate the functional integrity of inferior laryngeal nerve and the EBSLN. Although, postoperative video-strobolaryngoscopy and laryngeal EMG showed no evidence of inferior laryngeal nerve or EBSLN injury, the patients experienced swallowing impairments at the first postoperative month. The authors suggested that post-thyroidectomy swallowing problems likely originated from postoperative tissue fibrosis, emotional reasons, or postoperative pain when no objective evidence of RLN or SLNEB injury exists.

In our study, we used IONM during thyroidectomy in all patients. The predissection and postdissection EMG data showed no significant difference in the whole group. No LOS was encountered, and normal vocal cord function was assessed by postoperative laryngoscopy in all patients. However, reversible adverse EMG changes and CE were observed in eight and two patients, respectively. The percentage of postoperative decrease in UESRP was lower in patients with adverse EMG changes and CE than in those without such EMG parameters. EMG changes in EBSLN’s had no correlation with postoperative UESRP changes. Since the UESRP is primarily generated by the cricopharyngeus muscle, alterations in EMG signals during thyroidectomy may reflect impaired neural input to this muscle. Paradoxically, reduced innervation may not always result in hypotonia; in some patients, compensatory hypertonicity or discoordinated relaxation of the cricopharyngeus has been described (40, 41). Alternatively, inter-individual variability in postoperative neuromuscular adaptation could account for these heterogeneous responses, with some patients demonstrating marked UESRP reduction while others show stability or even increases. Unmeasured factors such as subtle anatomical differences or altered proprioceptive feedback may also have contributed. Overall, these findings indicate that the interaction between intraoperative EMG alterations and postoperative UESRP dynamics is not linear but multifactorial, and should be interpreted with caution until validated in larger series.

After thyroidectomy, 14% of our patients experienced new-onset dysphagia, and the decrease in UESRP was greater in these patients than in those without any postoperative change in dysphagia status. There was no evidence of adverse IONM parameters in patients with *de novo* dysphagia. Our study results indicate that impairment of swallowing and UES function following thyroidectomy may have multiple causes and can occur despite intact RLN and EBSLN function. However, our results suggest that postoperative onset of dysphagia is closely related to the reduction rate in UESRP. Although the reduction in UESRP appeared more prominent in patients with new-onset dysphagia, this finding did not reach conventional statistical significance (p=0.06) and should therefore be interpreted with caution. Nevertheless, the observed trend suggests a possible relationship that warrants confirmation in larger, adequately powered cohorts. Therefore, patients scheduled for thyroidectomy should be informed of potential post-thyroidectomy swallowing difficulties, even without nerve injury confirmed by IONM.

Our study has several limitations. First, the overall sample size was relatively small and the distribution of patients among the lobectomy, TTx, and TTx+CLND subgroups was unequal, which reduces the statistical power of subgroup analyses and limits the generalizability of the results. A further limitation is the absence of a validated swallowing questionnaire. While numerous prior studies have assessed dysphagia using subjective scales, only a limited number have applied objective HRM techniques. Our primary aim was to investigate whether intraoperative EMG changes could be predictive of postoperative UESRP alterations; however, the lack of a standardized patient-reported outcome measure restricts the ability to fully capture the patient’s experience of dysphagia. Despite these limitations, HRM provided an objective and gold-standard assessment of esophageal motility, and the use of routine intraoperative neuromonitoring and postoperative laryngoscopy strengthens the reliability of our findings. Future studies integrating both subjective and objective approaches in larger, multicenter cohorts would provide a more comprehensive understanding of postoperative swallowing dysfunction.

## Conclusion

Our study demonstrated that UESRP decreases after thyroidectomy even in patients without RLN or EBSLN injury confirmed by IONM and postoperative laryngoscopy. Patients with new-onset dysphagia tended to show a more pronounced reduction in UESRP, supporting the hypothesis that esophageal motility changes may contribute to postoperative swallowing difficulties. Larger thyroid size and volume were also associated with greater UESRP reduction, particularly in total thyroidectomy cases. Interestingly, patients with intraoperative adverse EMG changes exhibited less pronounced UESRP reduction, suggesting complex neuromuscular adaptations at the level of the cricopharyngeus muscle. The etiology of post-thyroidectomy dysphagia remains multifactorial and incompletely understood, and future studies combining objective manometry with validated patient-reported outcomes are needed to provide a more comprehensive understanding.

## Data Availability

The original contributions presented in the study are included in the article/**Supplementary Material**. Further inquiries can be directed to the corresponding author.
