# Supplementary material for: Influence of intraoperative electromyographic changes and surgical extent on post-thyroidectomy esophageal motility: a prospective cohort study
Source: Front Endocrinol (Lausanne). 2025 Oct 6;16:1651936. doi: 10.3389/fendo.2025.1651936 (PMC12535883; doi:10.3389/fendo.2025.1651936)
Supplement: Supplementary file 1 [file Table1.docx]

**Supplementary Table 1.** IONM EMG data before and after thyroidectomy in lobectomy, TT, and TT+CLND groups

|  | L | p | TT | p | TT+CLND | p |
| --- | --- | --- | --- | --- | --- | --- |
| *Right Vagus* |  | | | | | |
| V1 (µV) | 400  (290-510) |  | 430  (170-1930) |  | 520  (200-1000) |  |
| V2 (µV) | 390  (340-470) | 1 | 360  (90-1920) | 0,12 | 530  (350-960) | 0,37 |
| L1 (mS) | 4,2(3,2-4,5) |  | 4(3,3-6,2) |  | 3,9(2,5-5) |  |
| L2 (mS) | 5,2(3-5,7) | 0,28 | 4(3,5-6,2) | 0,6 | 3,8(2,4-6) | 0,86 |
| *Right RLN* |  | | | | | |
| R1 (µV) | 520  (410-580) |  | 440  (200-1360) |  | 555  (230-1030) |  |
| R2 (µV) | 440  (340-530) | 1 | 480  (190-1550) | 0,27 | 590  (310-920) | 0,34 |
| L1 (mS) | 2(1,7-2,1) |  | 1,8(1,4-2,5) |  | 2,2(1,6-2,7) |  |
| L2 (mS) | 3,4(1,4-3,5) | 0,28 | 1,8(1,5-2,4) | 0,97 | 1,9(1,4-2,3) | 0,14 |
| *Left Vagus* |  | | | | | |
| V1 (µV) | 380  (230-1000) |  | 590  (90-1410) |  | 380  (190-1060) |  |
| V2 (µV) | 770  (250-1060) | 0,5 | 430  (60-1220) | 0,43 | 415  (80-980) | 0,35 |
| L1(mS) | 6,1(5,7-7,6) |  | 6,8(4,8-7,7) |  | 5,9(1,5-6,5) |  |
| L2(mS) | 6(5,7-7,6) | 0,1 | 6,6(5,1-7,6) | 0,95 | 6,2(5,1-7,6) | ***0,04**** |
| *Left RLN* |  | | | | | |
| R1 (µV) | 1420  (370-1820) |  | 610  (40-2460) |  | 535  (50-1500) |  |
| R2 (µV) | 1200  (640-1670) | 0,89 | 500  (130-2180) | 0,24 | 535  (50-1420) | 0,80 |
| L1(mS) | 1,9(1,6-2,4) |  | 2,3(1,4-2,9) |  | 2,2(1,5-3,8) |  |
| L2(mS) | 2,2(1,7-2,5) | 0,19 | 2(1,5-3,4) | ***0,027**** | 2,3(1,2-3,8) | 0,62 |

L: Lobectomy TT: Total thyroidectomy, CLND: Central lymph node dissection, RLN: Recurrent laryngeal nerve, μV: microvolt, mS: millisecond

**Supplementary Table 2.** Percentage Change in IONM-EMG According to Surgical Extent – 1

| Change rate% | L vs TT | p | L vs TT+CLND | p |
| --- | --- | --- | --- | --- |
| *Right Vagus* |  | | | |
| Amplıtude | 4(-3 ̶ 17) vs -5(-56 ̶ 27) | 0,66 | 4(-3 ̶ 17 vs 5,8(-33 ̶ 75) | 0,52 |
| Latency | 7,7(-6 ̶ 35) vs 0(-19 ̶ 15) | 0,26 | 7,7(-6 ̶ 35) vs -1,3(-23 ̶ 87) | 0,31 |
| *Right RLN* |  | | | |
| Amplıtude | 0,96(-41 ̶ 7) vs  3,6(-26 ̶ 274) | 0,30 | 0,96(-41 ̶ 7) vs  8,9(-38 ̶ 82) | 0,13 |
| Latency | 30(-17 ̶ 75) vs  0(-40 ̶ 64) | 0,41 | 30(-17 ̶ 75) vs  -4,1(-48 ̶ 31) | 0,17 |
| *Left Vagus* |  | | | |
| Amplıtude | 32(-23 ̶ 242) vs  -13(-57 ̶ 66) | 0,35 | 32(-23 ̶ 242) vs  -8,6 (-64 ̶ 88) | 0,32 |
| Latency | -2,3(-3,4 ̶ 0) vs  -1,3(-16 ̶ 11) | 0,49 | -2,3(-3,4 ̶ 0) vs  3,3(-1,5 ̶ 266) | ***0,001**** |
| *Left RLN* |  | | | |
| Amplıtude | -12(-19 ̶ 31) vs  14(-54 ̶ 225) | 0,49 | -12(-19 ̶ 31) vs  1,8 (-78 ̶ 283) | 0,95 |
| Latency | 12(-5 ̶ 37) vs  -10(-26 ̶ 17) | ***0,01**** | 12(-5 ̶ 37) vs  6,8(-50 ̶ 133) | 0,79 |

L: Lobectomy TT: Total thyroidectomy, CLND: Central lymph node dissection, RLN: Recurrent laryngeal nerve

**Supplementary Table 3.** Percentage Change in IONM-EMG According to Surgical Extent – 2

| Change rate% | TT vs TT+CLND | p |
| --- | --- | --- |
| *Right Vagus* |  | |
| Amplıtude | -5(-56 ̶ 27) vs 5,8(-33 ̶ 75) | 0,08 |
| Latency | 0(-19 ̶ 15) vs -1,3(-23 ̶ 87) | 0,98 |
| *Right RLN* |  | |
| Amplıtude | 3,6(-26 ̶ 274) vs  8,9(-38 ̶ 82) | 0,75 |
| Latency | 0(-40 ̶ 64) vs-4,1(-48 ̶ 31) | 0,27 |
| *Left Vagus* |  | |
| Amplıtude | -13(-57 ̶ 66) vs  -8,6 (-64 ̶ 88) | 0,75 |
| Latency | -1,3(-16 ̶ 11) vs  3,3(-1,5 ̶ 266) | ***0,009**** |
| *Left RLN* |  | |
| Amplıtude | 14(-54 ̶ 225) vs  1,8 (-78 ̶ 283) | 0,32 |
| Latency | -10(-26 ̶ 17) vs  6,8(-50 ̶ 133) | 0,12 |

TT: Total thyroidectomy, CLND: Central lymph node dissection, RLN: Recurrent laryngeal nerve

**Supplementary Table 4. Clinical and pathological characteristics**

|  |  |  |  |  |
| --- | --- | --- | --- | --- |
| Papillary thyroid cancer n (%)  Papillary microcarcinoma n(%) |  |  |  | 29 (80,5)  13 (45) |
| Thyroiditis [n(%)] |  |  |  | 13 (36) |
| Tumor diameter (cm) |  |  |  | 1,3±0,96 |
| Dominant nodule diameter (cm) |  |  |  | 2,37±1,3 |
| Volume cm^3^  Total thyroid volume  Right lobe volume  Left lobe volume |  |  |  | 22 (10-458)  11,9 (4,5-258)  11 (4,3-200) |

|  |
| --- |

**Supplementary Table 5. A comparison of changes in upper oesophageal sphincter resting pressure (UESRP) according to the extent of surgery.**

|  | Percentage change in UESRP | p |
| --- | --- | --- |
| L | -8,7 (-82 ̶ 32) |  |
| TT | -44 (-86 ̶ 135) |  |
| TT+CLND | -16 (-80 ̶ 66) |  |
| L vs TT |  | 0,38 |
| TT vs TT+CLND |  | ***0,032**** |
| L vs TT+CLND |  | 0,5 |

L: Lobectomy TT: Total thyroidectomy, CLND: Central lymph node dissection
